# Endoscopic Characterization of Giant Choledocholithiasis and Its Correlation With Primary Choledocholithiasis

**DOI:** 10.7759/cureus.64956

**Published:** 2024-07-19

**Authors:** Marisol Ramos Portales, Carlos Martínez Álvarez, José P Salcedo Gómez, Ericka L Tadeo Hernández, Luis J Sánchez Fonseca, Monserrat Tapia Macías, Ana K Partida Montes, Ana S González Izaguirre, María F González Castillo, Juan C Sainz Hernández

**Affiliations:** 1 General Surgery, Institute for Social Security and Services for State Workers Regional Hospital, Leon, MEX; 2 Endoscopy, Institute for Social Security and Services for State Workers Regional Hospital, Leon, MEX; 3 Endoscopy, General Hospital of Mexico "Dr. Eduardo Liceaga", Mexico City, MEX; 4 General Surgery, Central Hospital "Dr. Ignacio Morones Prieto", San Luis Potosi, MEX; 5 General Medicine, Autonomous University of San Luis Potosi, San Luis Potosi, MEX; 6 General Surgery, Institute for Social Security and Services for State Workers General Hospital, San Luis Potosi, MEX; 7 Colon and Rectal Surgery, Institute for Social Security and Services for State Workers Regional Hospital, Leon, MEX

**Keywords:** endoscopic retrograde cholangiopancreatography, acute cholangitis, common bile duct, primary choledocholithiasis, giant choledocholithiasis

## Abstract

Introduction: Gallstone disease is extremely prevalent in Western society, and the prevalence of common bile duct (CBD) stones with concomitant cholelithiasis increases significantly in the elderly. Different variants influence the treatment of this pathological entity, such as the origin of the stones, their location and quantity, comorbidities of the patient, impaction, and size of the lithos, the latter being an independent predictive factor. In most situations, choledocholithiasis can be resolved with endoscopic retrograde cholangiopancreatography (ERCP); however, in complex cases, such as giant choledocholithiasis (GC), advanced surgical, endoscopic, and percutaneous techniques are required to remove gallstones. The main objective was to determine if there is a correlation between GC and primary choledocholithiasis (PC). The secondary objective consisted of describing the endoscopic characterization of GC.

Methods: The present study is a cross-sectional and single-center study. The study population consisted of patients of the Institute for Social Security and Services for State Workers (ISSSTE by its acronym in Spanish) Regional Hospital, León, Guanajuato, belonging directly to this center or referred, who required medical attention by the General Surgery/Endoscopy Service with the diagnosis of choledocholithiasis, during the period between January 2017 and December 2022. The Kolmogorov-Smirnov test was used as the normality test. Quantitative variables were reported as mean and standard deviation if the data distribution was normal, in contrast with the expression of data in the median and interquartile range if an abnormal distribution was found. Moreover, the qualitative variables are reported in frequencies or percentages. The Chi-square test was performed as the independence test. The significance level was a 95% confidence interval (p-value 0.05). The effect size was calculated with the odds ratio (OR).

Results: Out of a total of 177 patients, 33 corresponded to PC (18.6%), and 144 belonged to the secondary choledocholithiasis (SC) group (81.4%). Likewise, regarding the dimensions of the lithos, 59 patients (33.3%) presented GC and 118 (66.7%) presented non-GC. Among the 59 patients with GC, 36 were female (61%) and 23 were male (39%). Regarding age, the distribution was as follows: mean 62 ± 12 years, with a minimum value of 29 and a maximum of 88 years. The non-parametric test used to determine the existence or not of a correlation between the variables was Pearson's Chi-square, whose value was 60.509, with a p < 0.001, demonstrating the presence of a correlation between PC and GC. The effect size was corroborated and defined with the OR, whose value was 39.6 (confidence interval (CI) 11.308-139.069).

Conclusions: There is a significant correlation between GC and PC, and it was found that mechanical lithotripsy was the most performed initial extraction method for GC; furthermore, a higher rate of complete endoscopic resolution was found, as well as no complications related to the procedure, which contrasts with the literature. It would be interesting to use the information revealed in the present study as a landmark in future research in this regard.

## Introduction

Gallstone disease is extremely prevalent in Western society. The prevalence of common bile duct (CBD) stones with concomitant cholelithiasis increases with age from 8% to 15% in patients <60 years and up to 60% in the elderly [1,2]. In Mexico, the prevalence of biliary disease is 8.5% and 20.5% in men and women, respectively. In patients over 60 years of age, it may represent up to 30% of surgical indications, both emergency and elective [3]. 

Laparoscopic cholecystectomy (LC) is widely accepted as the first choice for the treatment of symptomatic gallstones with the exception of specific situations, such as certain anatomical variations [4], or comorbidities. However, when cholelithiasis and choledocholithiasis are concomitant, their management is controversial for both surgeons and endoscopists [5]. It is estimated that between 3.4% and 10% of patients have choledocholithiasis at the time of cholecystectomy. The frequency of choledocholithiasis after cholecystectomy ranges from 1.2% to 14%, although only 0.3% of patients will present symptoms [6].

Choledocholithiasis can be primary (PC) (stones originally formed in the bile duct) or secondary (SC) (stones that have migrated from the gallbladder to the CBD). PC accounts for 4-14%, made by dark-brown calcium bilirubinate stones, whereas in SC, which accounts for 86-96%, the stones are usually assembled by cholesterol [6].

PC was defined in 1977 by Saharia et al. as occurring two years or more after cholecystectomy (with or without bile duct exploration) in patients without an elongated cystic stump or bile duct stricture [7]. It has been described even 33 years after cholecystectomy and in patients with gallbladder agenesis. PC appears to be more common in Asia than in Western countries. Treatment is always challenging due to high recurrence (up to 41.7%) [6].

Among the factors associated with the development of biliary lithiasis are female gender, pregnancy, oral contraceptives, hormone replacement therapy, diabetes mellitus, obesity, hypercholesterolemia, hypertriglyceridemia, low high-density lipoprotein cholesterol, recurrent cholangitis, surgery with resection of the terminal ileum, sudden or progressive weight loss, some medications such as third-generation cephalosporins, and hemolytic anemia. Likewise, the presence of the juxtapapillary duodenal diverticulum appears as a risk factor for the development of PC; this is not the case for SC [3,8]. Some other risk factors have also been identified, such as advanced age, abnormal biliary structure, the number of liths, and biliary infection (*Enterobacter* and *Helicobacter pylori*) [9]. The presence of two or more CBD stones, cholesterol stones, and acute bile duct angulation (<145°) are associated with recurrent CBD stones after cholecystectomy [10]. 

The recurrence rate of choledocholithiasis after endoscopic retrograde cholangiopancreatography (ERCP) is 2-22%. Age older than 65 years, history of choledocholithotomy, endoscopic papillary balloon dilatation, endoscopic sphincterotomy, CBV stent implantation, multiple ERCP procedures (≥2), intrahepatic bile duct lithiasis, periampullary diverticula, choledocholithiasis of diameter ≥10 mm, bile duct-duodenum fistula, biliary tract infection, and failure to administer presurgical antibiotics have been found to be independent risk factors for the recurrence of choledocholithiasis after ERCP [11].

The main objective of this study was to determine if there is a correlation between GC and PC. The secondary objective consisted of describing the endoscopic characterization of GC.

## Materials and methods

The present study is a cross-sectional and single-center study. The study population consisted of patients of the Institute for Social Security and Services for State Workers (ISSSTE by its acronym in Spanish) Regional Hospital, León, Guanajuato, belonging directly to this center or referred, who required medical attention by the General Surgery/Endoscopy Service during the period between January 2017 and December 2022. The Research Ethics Committee of the ISSSTE Regional Hospital of León, Guanajuato, through the University of Guanajuato, issued the approval on May 11, 2023 with legal document ID HRL/CEI/JI/088/2023 based on the CONBIOÉTICA-11-CEI-001-20230127 registry and in accordance with the Declaration of Helsinki of the World Medical Association.

The inclusion criteria were made up of the following: patients with a diagnosis of choledocholithiasis confirmed by ERCP, whether it was the first event or a recurrence, of any age over 18 years, with or without a history of cholecystectomy, and for whom all the elements in the clinical record were complete. The exclusion criteria were as follows: patients in whom despite the probable diagnosis of choledocholithiasis according to the criteria of the American Society for Gastrointestinal Endoscopy 2019, it was ruled out by ERCP; post-surgical, inflammatory or tumor stenosis of the biliary tract, primary sclerosing cholangitis; gallbladder cancer, common bile duct cysts, cirrhosis or other liver diseases, tumors of the periampullary area, chronic pancreatitis of the pancreatic head, and Lemmel syndrome (obstructive jaundice due to periampullary duodenal diverticulum) in the absence of choledocholithiasis. Elimination criteria were determined by the following: patients with incomplete diagnostic protocol and loss of follow-up.

**Figure 1:**
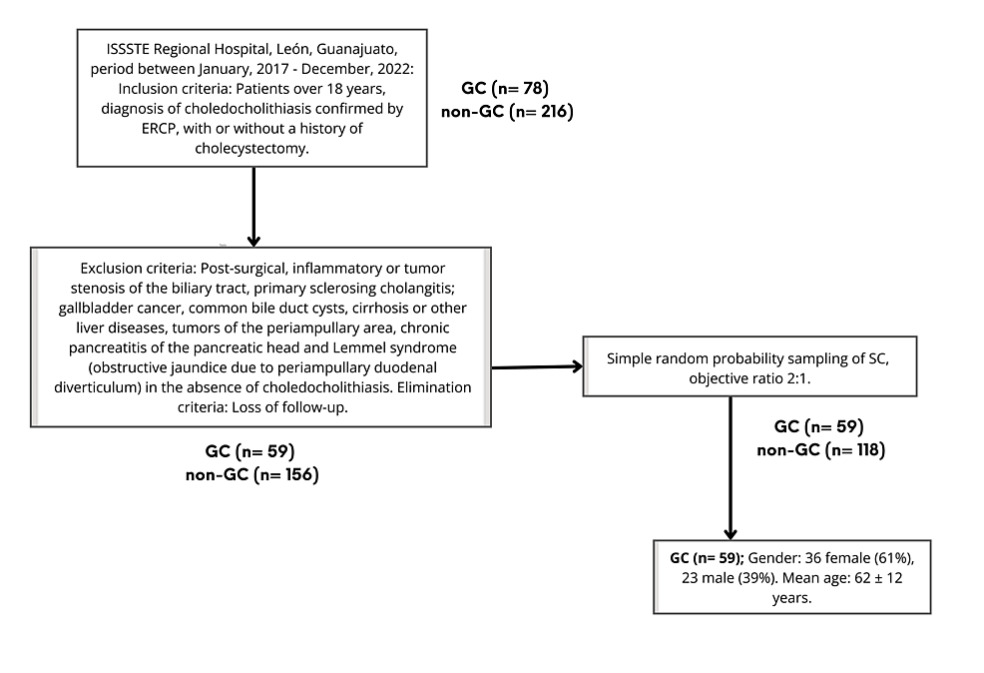
Method flowchart. ISSSTE: Institute for Social Security and Services for State Workers, GC: giant choledocholithiasis, non-GC: non-giant choledocholithiasis, ERCP: endoscopic retrograde cholangiopancreatography, SC: secondary choledocholithiasis

All patients who met the inclusion criteria and not exclusion criteria in the period between January 2017 and December 2022 were included, and they were then divided into two groups (GC and non-GC). Choledocholithiasis with at least one lith with one of the dimensions ≥2 cm was considered a giant lithiasis. Of the total 78 patients with GC, only 59 were included due to reiterative cases or those with exclusion criteria. Likewise, of the total 216 patients with non-GC, 60 were initially excluded due to the exclusion criteria, and 118 patients were subsequently selected according to simple random probability sampling, with the objective of maintaining a ratio of 2:1 (Figure 1). 

The measured variables were made up of the following: gender, age, PC, SC, juxtapapillary duodenal diverticulum, extrahepatic bile duct diameter, number of lithos, lith size, endoscopic resolution, extraction method, biliary prosthesis, and complications (Table 1). 

**Table 1:** Operational definition of variables. ERCP: endoscopic retrograde cholangiopancreatography

Variable	Definition	Unit of measure
Gender	Identity assigned at birth	Male/Female
Age	Years of life of a person	Years
Primary choledocholithiasis	Choledocholithiasis one year after cholecystectomy	Yes/No
Secondary choledocholithiasis	Choledocholithiasis in the absence of cholecystectomy or within one year after cholecystectomy surgery.	Yes/No
Juxtapapillary duodenal diverticulum	Duodenal diverticulum adjacent to ampulla of Vater	Yes/No
Extrahepatic bile duct diameter	Dimension of the extrahepatic bile duct lumen measured by cholangiography during ERCP.	Millimeters
Number of liths	Number of lithos in the common bile duct	Arabic numeral
Lith size	Dimension of the major axis of the lith	Millimeters
Endoscopic resolution	Endoscopic removal of common bile duct stones corroborated by cholangiography during ERCP.	Yes/No
Extraction method	Technique for the removal of lithos in the common bile duct	Precut papillotomy/sphincterotomy/balloon catheter/basket/lithotripsy
Biliary prosthesis	Plastic or metallic bile duct stents	Yes/No
Complications	Bleeding or perforation of the biliary or intestinal tract	Yes/No

Statistical analysis was performed in IBM SPSS Statistics for Windows, Version 29.0.2.0 (released 2023, IBM Corp., Armonk, NY).

The Kolmogorov-Smirnov test was used as the normality test. Quantitative variables were reported as mean and standard deviation if the data distribution was normal, in contrast with the expression of data in the median and interquartile range if an abnormal distribution was found. Moreover, the qualitative variables are reported in frequencies or percentages.

The Chi-square test was performed as the independence test. The effect size was calculated with the OR. The significance level was a 95% confidence interval (CI) (p-value < 0.05).

## Results

Out of a total of 177 patients, 59 patients (33.3%) presented GC and 118 (66.7%) corresponded to non-GC. Likewise, regarding the origin, 33 corresponded to PC (18.6%), and 144 belonged to the SC group (81.4%) (Table 2). The non-parametric test used to determine the existence or not of a correlation between the variables was Pearson's Chi-square, whose value was 60.509, with a p-value <0.001, demonstrating the presence of a correlation between PC and GC (Tables 3-4). 

**Table 2:** Distribution of choledocholithiasis according to the origin and lith size. N: sample size

Classification	Variable	N	%	Total
Lith size	Giant choledocholithiasis	59	33.3%	177 (100%)
	Not giant choledocholithiasis	118	66.7%	
Origin	Primary choledocholithiasis	33	18.6%	177 (100%)
	Secondary choledocholithiasis	144	81.4%	

**Table 3:** Cross-tabulation of origin versus dimensions of choledocholitiasis. * Significant p-value

Origin		Dimensions		Total
		Giant	Not giant	
Primary	Count	30	3	33
	Expected count	11.0	22.0	33.0
Secondary	Count	29	115	144
	Expected count	48.0	96.0	144.0
Total	Count	59	118	177
	Expected count	59.0	118.0	177.0
p value		<0.01*		

**Table 4:** Chi-square tests. N: sample size, gl: degrees of freedom. * Significant p-value.

Chi-square tests	Value	gl	Asymptotic significance (bilateral)	Exact significance (bilateral)	Exact significance (unilateral)
Pearson's Chi-square	60.509	1	< 0.001>*	-	-
Continuity correction	57.366	1	< 0.001>	-	-
Likelihood ratio	60.551	1	< 0.001>	-	-
Fisher's exact test	-	-	-	< 0.001>	< 0.001>
N of valid cases	177	-	-	-	-

The effect size was corroborated and defined with the OR, whose value was 39.6 (CI 11.308-139.069) (Table 5). 

**Table 5:** Odds ratio value.

Risk estimate	Value	95% Confidence interval	
		Lower	Upper
Odds ratio for PRIMARY (Yes / No)	39.655	11.308	139.069
For cohort GIANT = Yes	4.514	3.204	6.359
For cohort GIANT = No	0.114	0.039	0.336
N of valid cases	177		

In addition, it was found that of the 59 patients with GC, 36 were female (61%) and 23 were male (39%). Regarding age, the distribution was as follows: mean 62 ± 12 years, with a minimum value of 29 and a maximum of 88 years.

Likewise, it was found that nine patients had a juxtapapillary duodenal diverticulum (15.3%), of which four corresponded to intradiverticular papilla (6.8%); on the other hand, 50 patients (84.7%) did not present this characteristic.

**Figure 2:**
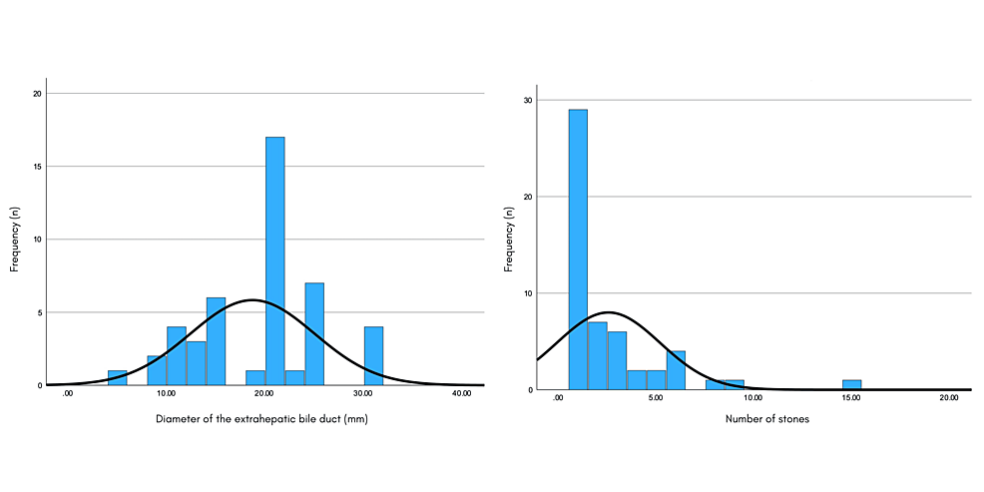
Distribution of the diameter of the extrahepatic bile duct and number of stones in GC. GC: giant choledocholithiasis

The diameter of the extrahepatic bile duct showed the following distribution: mean 18.69 ± 6.29 mm, with a minimum value of 5 and a maximum of 30. In relation to the number of lithos found, the following distribution was shown: mean 2.54 ± 2.64, with a minimum value of 1 and a maximum of 15 (Figure 2). 

**Figure 3:**
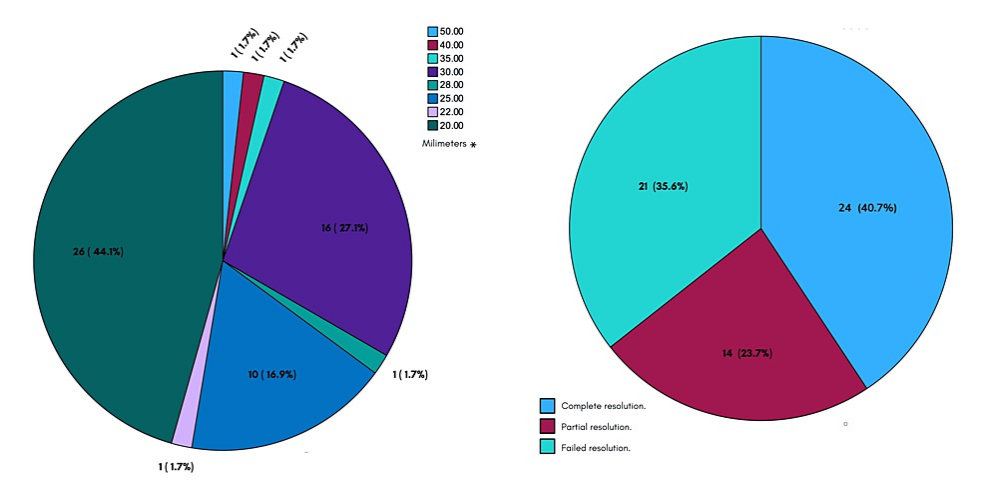
Frequencies per stone size and endoscopic resolution frequencies in GC. GC: giant choledocholithiasis. * Size not specified in two cases.

The stone size was as follows: mean 25 ± 6 mm, with a minimum value of 20 and maximum of 50. Complete endoscopic resolution was achieved in 40.7% of the cases (24 patients) and partial resolution (incomplete extraction of the totality of the lithos or its fragments) in 23.7% (14 patients), and the resolution was unsuccessful in 35.6% of the cases (21 patients) (Figure 3). 

**Figure 4:**
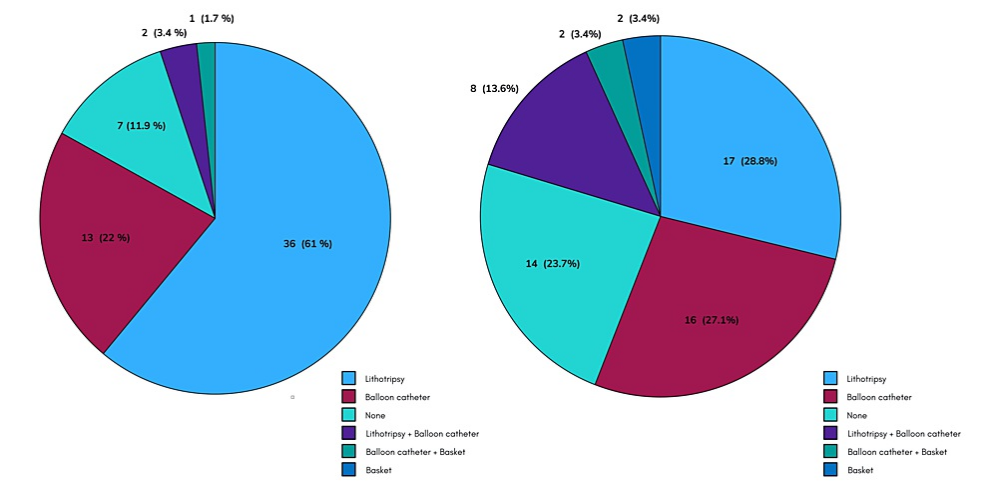
Bile duct endoscopic access technique and extraction method distribution in GC. GC: giant choledocholithiasis

In terms of the extraction methods used, the distribution was as follows: precut papillotomy 3.4% (two patients), sphincterotomy 61% (36 patients), precut papillotomy plus sphincterotomy 11.9% (seven patients), and sphincteroplasty 1.7% (one patient). Moreover, lithotripsy was performed in 28.8% (17 patients), balloon catheter in 27.1% (16 patients), basket in 3.4% (two patients), lithotripsy plus balloon catheter in 13.6%, and balloon catheter plus basket in 3.4% (two patients) (Figure 4). 

Similarly, the biliary prosthesis was placed in 19 patients (32.2%), it was replaced in 15 patients (25.4%), and it was not required in 25 patients (42.4%). Finally, there were no complications related to ERCP in the group of patients with GC.

## Discussion

GC constitutes a different section within choledocholithiasis, mainly when it refers to its treatment; likewise, PC and SC do not constitute a homogeneous group.

PC has been identified as an infection as part of the etiology [12]. Moreover, two significant risk factors for the recurrence of PC after endoscopic sphincterotomy have been identified: bile duct diameter ≥13 mm after stone removal and the location of the papilla at the inner edge or deep to a duodenum diverticulum [13]. Likewise, the incidence of gallstones is significantly higher in the presence of periampullary diverticula, and when considering the site and origin of gallstones, the association between diverticula and gallstones is significant in patients with PC, but not with SC [8]. In addition, it has been shown that the vast majority of patients (80%) with choledocholithiasis after cholecystectomy will present the disease within three years after surgery [1].

Contemplating GC, its characterization has been a complicated issue, and despite its low incidence, it represents a variety of implications for both, patients and health systems, which are not easy to ignore. Recently, Dai et al. published a study focused on the characteristics of biliary microbiota in patients with GC, as one more step in the knowledge of this entity [14]. GC isolated events have also been reported, as is the one published by Kumar et al. secondary to long-dwelling biliary endoprosthesis [15].

ERCP is the cornerstone for the treatment of CBD stones, being capable of treating 90% of choledocholithiasis [16]. However, there are complications related to ERCP, which seem to be more frequent in centers that perform less than 200 ERCPs per year [17,18]. One of the most serious post-ERCP complications is pancreatitis. Post-sphincterotomy bleeding has also been reported in up to 2% of cases, which is immediate in up to 30% of patients; late bleeding can occur up to two weeks after the procedure [19]. It is worth mentioning that no complications occurred during ERCP in GC in our study, differing from data reported in previous studies, despite being widely known that complications are more likely to occur in GC. The choice of sequential treatment for CBD lithos is influenced by several factors including the availability of technical expertise, instrumentation, and financial reimbursement [20]. 

In accordance with the European Society of Gastrointestinal Endoscopy (ESGE), after endoscopic biliary sphincterotomy, bile duct stones must be removed with a balloon as the first-line approach due to a 30-50% reduction of the use of mechanical lithotripsy, reserving mechanical lithotripsy in the case of failure [21]. The approach of GC in our center is mostly in accord with these guidelines, such that sphincterotomy was performed in 61% (36 patients); however, the balloon catheter was used only in 30.5% (18 patients) initially, lithotripsy being in turn considerably used forming 42.4% (25 patients). Randomized controlled trials have compared balloon over cannula techniques for small bile duct stones (<11 mm), reporting slightly better results for balloon over cannula, especially for stones <6 mm. While most cases are successfully treated with these approaches, about 10-15% of them require alternative and/or adjunctive techniques to achieve bile duct clearance. These lithos are defined as "difficult" bile duct stones, a broad category of cases that encompasses very diverse scenarios, including large, multiple, peculiarly shaped stones, located over a stricture or impacted, intrahepatic, altered distal bile duct pathway, juxtapapillary duodenal diverticulum, surgically altered anatomy, or patient-specific general conditions [22]. Hence, it is worth highlighting the lower failure rate of endoscopic complete resolution in patients with GC reported in our center (59.35%), taking into account our cut-off point of lithos ≥20 mm, contrasting with a failure rate of up to 88% reported by Corona et al., even when the stones measure between 1 and 1.5 cm [3]. In addition, the ESGE mentions that stones larger than 15 mm usually increase the difficulty of stone extraction [21].

Mechanical lithotripsy with an extraction basket is the most widely used lithotripsy technique; when this fails, extracorporeal shock wave lithotripsy could represent an alternative. The innovative techniques related to lithotripsy assisted by cholangioscopy with laser or electrohydraulic system have significantly improved the management of complex cases of choledocholithiasis; however, GC removal by direct cholangioscopy with a standard gastroscope has been reported to provide greater stability and visibility, as well as strong irrigation and suction, allowing high-quality electrohydraulic lithotripsy. This technique should only be used for giant stones >2 cm and dilated bile ducts >15 mm [17]. Li et al. reported that the clinical efficacy of SpyGlass-guided laser lithotripsy for the treatment of large CBD stones is not inferior to that of laparoscopic common bile duct exploration, and it is also less invasive [23]. Otherwise, Maydeo et al. have considered evaluating cholangioscopy-assisted lithotripsy for primary use, rather than following a failed approach, in certain scenarios [24]. Acho et al. proposed the application of robotic choledochoscopy, showing, on one hand, the tendency toward technological advancement and, on the other, the high degree of complexity in treating "difficult" bile duct stones [25].

The present study determined the existence of a significant correlation between GC and PC, a characteristic that had not been previously established. Likewise, we report the description of the endoscopic characterization of a series of 59 patients with GC (≥20 mm), which is so far the largest reported in the literature reviewed, which will contribute in some way to improve the diagnostic and/or therapeutic approach algorithm for this complex entity. 

However, there were some limitations to this study, one of them being the low incidence of GC, which translates into the difficulty of obtaining a larger sample, as well as the cross-sectional nature of our study. Hence, it would be interesting to use the data obtained here as a watershed in future prospective, randomized, controlled trials.

## Conclusions

There is a significant correlation between GC and PC. It was found that mechanical litrotripsy was the most performed initial extraction method for GC. Furthermore, a higher rate of complete endoscopic resolution was found, as well as no complications related to the procedure, which contrasts with the literature.

Despite the evolution of medicine and the unquantifiable advance that the advent of endoscopy and especially the CPRE has represented in all aspects, there are currently multiple unresolved questions about the therapeutic algorithm to be adopted in cases of GC, which represents a real challenge due to the complexity faced by both surgeons and endoscopists when dealing with this entity, regarding the high level of technical difficulty for the extraction of lithos of such size. This is interesting in the context of the high prevalence of CBV lithiasis, as well as the technological revolution in terms of the possibilities of diagnosing and treating it. This study contributes to the characterization of this entity, and the fact of proving a correlation between GC and PC constitutes a crucial point to build the pillars of the improvement of its therapeutic approach in the future. It would be interesting to use the information revealed in the present study as a landmark in future research in this regard.
